# Identifying Differences between Greek Adolescent Suicide Attempters and Adolescent Patients with No Suicidal Behavior

**DOI:** 10.1192/j.eurpsy.2023.2375

**Published:** 2023-07-19

**Authors:** T. Tairi, N. Zilikis, F. Siamouli

**Affiliations:** 1 International Studies, American University of Sharjah, Sharjah, United Arab Emirates; 2 3rd Psychiatric Department, AHEPA General Hospital, Aristotle University of Thessaloniki, Thessaloniki, Greece

## Abstract

**Introduction:**

Youth suicide is a significant public health problem resulting in a major social and economic burden for communities and a devastating impact on families.

**Objectives:**

The present study, which is part of a continuing research on attempted suicide among adolescents conducted in Northern Greece since 1990, explored the circumstances and characteristics of adolescent suicide attempters, comparing them with a clinical sample of adolescents with no suicidal behavior.

**Methods:**

We retrospectively studied medical records and collected clinical data, sociodemographic and family characteristics of adolescents (all diagnoses except attempted suicide) referred to the Adolescent Unit of the AHEPA General Hospital for assessment and treatment between 2008 and 2018 (*N* = 160) and we compared them with our sample of adolescent suicide attempters (*N* = 182).

**Results:**

Of the 342 cases reviewed, 71.6% were female and aged 12 to 19 years (*M* = 15.39, *SD* = 1.81). Chi-square analyses showed that, compared with patients with no suicidal behavior, attempters were more frequently diagnosed with personality disorder and mood disorder. No differences were found between groups for substance-use disorder, psychosis, eating disorder and somatic symptom disorder. Attempters were also living in more problematic circumstances, such as such as severe family dysfunction and/or impairment, reported significantly more conflict with parents, had more school problems and had experienced romantic disappointment (see Table 1).

**Table 1.** Comparisons between suicide attempters and nonsuicidal comparison group on family psychosocial characteristics

	Suicide Attempters (*n =* 182)	Comparison Group (*n =* 160)			
	*n*	%	*n*	%	*χ*^2^
Malfunction/Inadequate family system	109	60.6	59	36.9	19.00***
School problems/difficulties	100	55.2	60	37.7	10.42***
Conflict with parents	97	53.9	66	41.3	5.42*
Romantic disappointment	41	22.9	10	6.3	18.34***
Conflict with siblings	26	14.4	12	7.5	4.11*
History of physical abuse/violence	25	14.0	16	10	1.25
History of sexual abuse – Rape	6	3.4	6	3.8	.04

**Conclusions:**

These findings highlight risk factors for suicide attempts and inform the development of suicide models that improve identification of adolescents at greatest risk to making a suicide attempt.

**Disclosure of Interest:**

None Declared

